# Association of Forced Vital Capacity with the Developmental Gene *NCOR2*

**DOI:** 10.1371/journal.pone.0147388

**Published:** 2016-02-02

**Authors:** Cosetta Minelli, Charlotte H. Dean, Matthew Hind, Alexessander Couto Alves, André F. S. Amaral, Valerie Siroux, Ville Huikari, María Soler Artigas, David M. Evans, Daan W. Loth, Yohan Bossé, Dirkje S. Postma, Don Sin, John Thompson, Florence Demenais, John Henderson, SpiroMeta consortium, CHARGE consortium, Emmanuelle Bouzigon, Deborah Jarvis, Marjo-Riitta Järvelin, Peter Burney

**Affiliations:** 1 Respiratory Epidemiology, Occupational Medicine and Public Health, National Heart and Lung Institute, Imperial College, London, United Kingdom; 2 Leukocyte Biology, National Heart and Lung Institute, Imperial College London, London, United Kingdom; 3 Mammalian Genetics Unit, MRC Harwell, Oxon, United Kingdom; 4 Respiratory Department, Royal Brompton and Harefield NHS Foundation Trust, London, United Kingdom; 5 Department of Epidemiology and Biostatistics, School of Public Health, Imperial College London, London, United Kingdom; 6 MRC-PHE Centre for Environment & Health, London, United Kingdom; 7 Univ. Grenoble Alpes, IAB, Team of Environmental Epidemiology applied to Reproduction and Respiratory Health, F-38000, Grenoble, France; 8 INSERM, IAB, Team of Environmental Epidemiology applied to Reproduction and Respiratory Health, F-38000, Grenoble, France; 9 CHU de Grenoble, IAB, Team of Environmental Epidemiology applied to Reproduction and Respiratory Health, F-38000, Grenoble, France; 10 Biocenter Oulu, University of Oulu, Oulu, Finland; 11 Genetic Epidemiology Group, Department of Health Sciences, University of Leicester, Leicester, United Kingdom; 12 University of Queensland Diamantina Institute, Translational Research Institute, Brisbane, Australia; 13 MRC Integrative Epidemiology Unit, University of Bristol, Bristol, United Kingdom; 14 Department of Epidemiology, Erasmus Medical Center, Rotterdam, The Netherlands; 15 Institut universitaire de cardiologie et de pneumologie de Québec, Department of Molecular Medicine, Laval University, Québec, Canada; 16 Department of Pulmonary Diseases, University of Groningen, University Medical Center Groningen, Groningen, The Netherlands; 17 The University of British Columbia Center for Heart Lung Innovation, St-Paul’s Hospital, Vancouver, Canada; 18 Department of Health Sciences, University of Leicester, Leicester, United Kingdom; 19 INSERM, UMRS-946, Genetic Variation of Human Diseases Unit, Paris, France; 20 Univ. Paris Diderot, Sorbonne Paris Cité, Institut Universitaire d’Hématologie, F-75007, Paris, France; 21 School of Social and Community Medicine, University of Bristol, Bristol, United Kingdom; 22 SpiroMeta consortium, Genetic Epidemiology Group, Department of Health Sciences, University of Leicester, Leicester, United Kingdom; 23 CHARGE consortium, Institutes of Health, Department of Health and Human Services, Research Triangle Park, North Carolina, United States of America; 24 Center for Life Course Epidemiology, Faculty of Medicine, P.O. Box 5000, FI-90014 University of Oulu, Oulu, Finland; 25 Unit of Primary Care, Oulu University Hospital, Kajaanintie 50, P.O. Box 20, FI-90220, Oulu, 90029 OYS, Finland; University Children's Hospital Basel, SWITZERLAND

## Abstract

**Background:**

Forced Vital Capacity (FVC) is an important predictor of all-cause mortality in the absence of chronic respiratory conditions. Epidemiological evidence highlights the role of early life factors on adult FVC, pointing to environmental exposures and genes affecting lung development as risk factors for low FVC later in life. Although highly heritable, a small number of genes have been found associated with FVC, and we aimed at identifying further genetic variants by focusing on lung development genes.

**Methods:**

Per-allele effects of 24,728 SNPs in 403 genes involved in lung development were tested in 7,749 adults from three studies (NFBC1966, ECRHS, EGEA). The most significant SNP for the top 25 genes was followed-up in 46,103 adults (CHARGE and SpiroMeta consortia) and 5,062 children (ALSPAC). Associations were considered replicated if the replication p-value survived Bonferroni correction (*p*<0.002; 0.05/25), with a nominal p-value considered as suggestive evidence. For SNPs with evidence of replication, effects on the expression levels of nearby genes in lung tissue were tested in 1,111 lung samples (Lung eQTL consortium), with further functional investigation performed using public epigenomic profiling data (ENCODE).

**Results:**

*NCOR2*-rs12708369 showed strong replication in children (p = 0.0002), with replication unavailable in adults due to low imputation quality. This intronic variant is in a strong transcriptional enhancer element in lung fibroblasts, but its eQTL effects could not be tested due to low imputation quality in the eQTL dataset. *SERPINE2*-rs6754561 replicated at nominal level in both adults (p = 0.036) and children (p = 0.045), while *WNT16-*rs2707469 replicated at nominal level only in adults (p = 0.026). The eQTL analyses showed association of *WNT16-*rs2707469 with expression levels of the nearby gene *CPED1*. We found no statistically significant eQTL effects for *SERPINE2*-rs6754561.

**Conclusions:**

We have identified a new gene, *NCOR2*, in the retinoic acid signalling pathway pointing to a role of vitamin A metabolism in the regulation of FVC. Our findings also support *SERPINE2*, a COPD gene with weak previous evidence of association with FVC, and suggest *WNT16* as a further promising candidate.

## Introduction

Forced vital capacity (FVC), a spirometric measure routinely used in clinical practice to approximate vital capacity, is increasingly recognised as an important parameter beyond its diagnostic and prognostic role in restrictive lung diseases. Unlike the ratio of forced expiratory volume in 1 second (FEV_1_) to FVC, an indicator of airway obstruction, FVC is a strong predictor of all-cause mortality in asymptomatic adults without chronic respiratory conditions[1]. Although the origins of a low FVC in the general population are poorly understood, there is a strong link to poverty[2], and in particular to low socio-economic status in early life[3]. Endemic vitamin A deficiency is associated with low FVC, and maternal supplementation with vitamin A before, during and after pregnancy, improves FVC in offspring[4]. Low FVC has also been associated with early exposure to particulate air pollution[5]. The deviation of an individual’s FVC values (and lung function in general) from the population mean has been shown to remain stable over time, with future values being predicted by early measurements (“tracking”)[6], which means that early life and genetic effects that manifest in childhood will influence the individual’s whole FVC life trajectory. Taken together, this evidence highlights the role of early life factors on adult FVC, which points to environmental exposures and genes affecting the development of the lung. Severe defects in lung development lead to neonatal death, but milder structural or functional defects could affect lung function and increase susceptibility to lung diseases that become clinically detectable during childhood or later life, including asthma and COPD[7]. This is supported by experimental work on *in-vitro* and animal models of lung function and disease[8].

Knowledge of the genetics of FVC is still limited. Biological candidates for FVC, mainly related to host defense, inflammatory pathway, pulmonary surfactant and oxidative stress, have been evaluated in candidate-gene association studies, but replication has been difficult. New candidates for FVC have been provided by genome-wide association (GWA) studies, the largest being a recent meta-analysis from the joint CHARGE and SpiroMeta consortia on 52,253 individuals, with replication of the top associations in 24,840 individuals[9]. It identified eight loci, of which six new (*EFEMP1*, *BMP6*, *MIR129-2-HSD17B12*, *PRDM11*, *WWOX*, *KCNJ2*), and two previously associated with FEV_1_ and FEV_1_/FVC (*GSTCD* and *PTCH1*). The eight loci explain 1.8% of FVC variation, and yet FVC heritability (proportion of FVC variation attributable to genetic factors) is estimated around 40–60% by familial aggregation and twin studies[10, 11] and, more recently, genome-wide data[12].

Available GWA datasets represent an invaluable resource to test hypotheses about the role of genetic pathways involved in specific pathophysiological mechanisms. We hypothesised that focusing on genes lying in pathways related to lung development could help identify new candidates for FVC and further our understanding of the underlying biological mechanisms.

## Materials and Methods

We evaluated the effect on FVC of 403 genes (24,728 SNPs) related to lung development in two stages. In Stage 1, all SNPs were tested for association with FVC in a meta-analysis of three European adult studies (ECRHS[13], NFBC1966[14], EGEA[15]). For replication in adults (CHARGE and SpiroMeta consortia)[9] and children (ALSPAC[16]) in Stage 2, we selected the best signal for the top 25 genes, defined as the SNP with the lowest meta-analysis p-value which satisfied the following criteria: minor allele frequency >0.05 and imputation quality (imputation R^2^) >0.7 in all three studies; low between-study heterogeneity defined as I^2^<30%, with I^2^ representing the percentage of total variation in effect estimates across studies due to heterogeneity rather than chance.

The rationale for limiting our replication analysis to the best signal for the top 25 genes was to maximise the probability of successful replication in children, where the sample size was only 5,062. With this sample size, testing for replication of 25 SNPs gives a power of about 80% to detect a variant explaining 0.3% of FVC residual variance, at a Bonferroni corrected p-value threshold of 0.002 (0.05/25). This assuming that genetic effects in children may be slightly stronger than in adults, where the variance explained by the eight loci previously identified[9] was 1.8%, an average of 0.23% per SNP.

### Selection of candidate genes and SNPs

Two experts in lung development, a basic scientist (C.H.D.) and a clinician scientist (M.H.), compiled a list of genes involved in lung development, first independently and then through agreement. The selection of genes was based on their knowledge of the topic, mainly using genetic evidence from animal models[8, 17, 18]. This initial list was extended to include additional genes suggested by: 1) pathways information obtained from KEGG[19]–relevant genes lying in the same pathways as those in the initial list; 2) information from published literature identified using HuGE Navigator[20]–genes considered as associated with lung development in previous genetic association studies. When in doubt about which genes to select from large gene families, those with higher gene expression in foetal lung were chosen, with information retrieved from the Human U133A/GNF1H Gene Atlas database using BioGPS[21].

The final list included 403 genes (S1 Table). According to NCBI gene definition, we retrieved SNPs within 2 kb upstream and 500 bp downstream of each gene, using the R package NCBI2R (http://cran.r-project.org/web/packages/NCBI2R). We identified 24,728 SNPs for which imputed data (based on HapMap release 22) were available for all three studies in Stage 1 (S1 Table).

### Study populations

#### Stage 1

Below and in Table 1 we briefly describe the three studies, with details on spirometry and genotyping methods summarised in S2 and S3 Tables.

**Table 1:** Characteristics of studies in Stage 1. N = number of subjects included in the analyses.

Study	N	Country	Sex[% male]	Age (years)		Height (cm)[Mean (SD)]	FVC (ml)[Mean (SD)]
				Absolute Range	Mean (SD)		
NFBC1966	5,218	Finland	47.9%	31–31	31 (0)	171.2 (9.2)	4,718 (987)
ECRHS	1,662	Spain, United Kingdom, France, Germany, Sweden, Norway, Switzerland, Estonia	47.5%	19.7–48.1	34.0 (7.1)	170.5 (9.5)	4,552 (1,031)
EGEA	869	France	46.1%	18.0–76.5	38.5 (12.6)	168.6 (8.5)	4,239 (982)

The Northern Finland Birth Cohort 1966 (NFBC1966) is a birth-cohort study in the provinces of Oulu and Lapland that recruited pregnant women with an expected date of delivery in 1966. A total of 12,231 children were recruited and followed-up in adulthood[14], with 6,033 participating in the clinical follow-up at 31 years. Of these, 5,218 individuals with GWA and spirometry data were included in this study.

The European Community Respiratory Health Survey (ECRHS) is an international cohort study designed to identify risk factors for asthma[13] that started in 1992–1994, with follow-up performed twice in the following 20 years. Included in this study are 1,662 subjects from the first survey (ECRHS I, age 20–48) with GWA and spirometry data available, recruited from 16 centres that used random sampling frameworks.

The Epidemiological study on the Genetics and Environment of Asthma (EGEA), which combines a case-control and a family-based study of asthma, was conducted in 1991–1995 (EGEA1), with follow-up after 12 years (EGEA2, 2003–2007)[15]. The study included 388 nuclear families, ascertained by one or two asthmatic adult or paediatric probands, and 415 population-based controls, totalling 2,120 subjects. This analysis only includes 869 non-asthmatic adults, using spirometry data from EGEA1 for subjects ≥18 year old at baseline and EGEA2 for those <18 in EGEA1.

#### Stage 2

The joint CHARGE (Cohorts for Heart and Aging Research in Genomic Epidemiology) and SpiroMeta consortia performed a GWA investigation of FVC in 52,253 individuals of European ancestry from 26 studies[9], which included ECRHS and NFBC1966. Included here are 46,103 individuals from 24 studies, after subtracting the contribution of ECRHS and NFBC1966. New effect estimates and standard errors were derived by taking a weighted difference between the original fixed-effect meta-analysis estimate and the pooled estimate of ECRHS and NFBC1966.

The Avon Longitudinal Study of Parents and their Children (ALSPAC) is a birth cohort study consisting initially of 14,541 women and their children recruited in the county of Avon, UK, in the early 1990s[16]. Included in this study are 5,062 white European children (50.3% male) of 8–9 years of age with GWA and spirometry data. Their mean height was 132.6 cm (standard deviation, SD: 5.8) and mean FVC 1,931 ml (SD: 319).

### Statistical analyses

#### Stage 1

Study-specific estimates for the three studies were obtained assuming an additive mode of inheritance. In ECRHS, linear regression analyses of the effects of the SNPs on FVC (in ml) were adjusted for age, age^2^, height, sex, centre, and first four ancestry principal components to control for residual population stratification. In NFBC1966, all subjects were 31 year olds and linear regression analyses were only adjusted for height, sex and first two principal components. In the family-based EGEA, the regression analyses were performed using linear mixed models to account for family structure, adjusting for age, age^2^, height, sex and first two principal components.

Inverse-variance weighted meta-analysis of the three studies using a fixed effect model was performed on a total of 7,749 individuals.

The association analyses for NFBC1966 were carried out using SNPTEST[22], while the analyses for ECRHS and EGEA and the meta-analysis were performed using R, version 3.0.1 (www.R-project.org).

#### Stage 2

Individual cohorts within CHARGE and SpiroMeta performed GWA analyses for FVC (ml) using linear regression adjusted for age, age^2^, height and sex (plus height^2^ and weight for CHARGE), as well as centre and/or principal components if appropriate[9].

In ALSPAC, linear regression analyses on FVC (ml) were performed adjusting for age, age^2^, height and sex. Principal components were not included since no evidence of population stratification was found in the study.

Replication of a SNP was defined based on evidence from Stage 2 only, rather than on combined evidence from Stage 1 and Stage 2, since this protects against the winner’s curse, an upwards bias typical of the screening stage[23]. We considered a SNP replicated if the effect estimate was in the same direction as in Stage 1 and the one-side p-value survived Bonferroni correction for multiple testing (*p*<0.002) in either adults or children. We considered replication evidence as suggestive if the p-value was significant only at nominal level.

### Lung eQTL data

For SNPs with evidence of replication, we investigated their effects on the expression of nearby genes (genes within 100 kb up and downstream from the SNP) in lung samples from the Lung QTL consortium. This includes data on 1,111 individuals undergoing lung surgery, recruited at Laval University (n = 409), University of British Columbia (n = 339) and University of Groningen (n = 363)[24].

Gene expression and genotyping profiles were obtained using a custom Affymetrix array (GEO platform GPL10379) and the Illumina Human1M-Duo BeadChip array, respectively. Expression values were extracted using the Robust Multichip Average method[25] implemented in the Affymetrix Power Tools software. Expression values were analysed with a robust regression model adjusted for age, sex and smoking status, using the R statistical package MASS (*rlm* function).

Genetic associations were performed in PLINK 1.9. A fixed-effect meta-analysis was used to pool the results across the three sites.

## Results

Stage 1 study-specific and meta-analysis results are reported in Table 2 for the best SNP of the top 25 genes, and in S1 Table for all 24,728 SNPs. Replication could only be performed for 24 SNPs, since no data were available for *EYA1* rs12549242 or any proxy (defined as a SNP with linkage disequilibrium, LD, R^2^>0.8) in CHARGE and SpiroMeta and ALSPAC. In Stage 2, one gene showed strong replication in children, *NCOR2*, with replication unavailable for adults due to low imputation quality; other two genes showed suggestive evidence of replication, one in both adults and children, *SERPINE2*, and the other in adults but not in children, *WNT16* (Table 3). The regional association plots for their lead SNP are presented in S1 Fig.

**Table 2:** Results for the best SNP of the top 25 genes in Stage 1: NFBC 1966, ECRHS, EGEA, and meta-analysis. Chr: chromosome; EA: effect allele; EAF: effect allele frequency, calculated as weighted average across the three studies; β (standard error, SE): estimate of the per-allele effect on FVC (ml); I^2^: magnitude of the between-study heterogeneity of effect estimates

SNP	Gene	Chr	Position	EA	EAF	NFBC1966(N = 5,218)			ECRHS(N = 1,662)			EGEA(N = 869)			Meta-analysis			
						β	SE	*P*	β	SE	*P*	β	SE	*P*	β	SE	*P*	I^2^ (%)
rs2820472	*WLS*	1	68,694,307	C	0.70	31.0	11.3	*0*.*0061*	30.3	21.7	*0*.*1621*	-17.5	32.0	*0*.*5851*	26.5	9.6	*0*.*0055*	4
rs832169	*PKP1*	1	201,256,771	A	0.17	29.2	15.8	*0*.*0646*	50.0	22.4	*0*.*0257*	41.1	31.9	*0*.*1984*	36.9	12.0	*0*.*0021*	0
rs7527525	*ACTN2*	1	236,902,560	C	0.33	20.0	11.7	*0*.*0875*	33.6	20.2	*0*.*0960*	63.7	28.0	*0*.*0238*	28.1	9.5	*0*.*0032*	8
rs3905417	*CTNNA2*	2	80,181,443	A	0.23	29.9	12.2	*0*.*0144*	30.4	24.4	*0*.*2127*	45.7	34.0	*0*.*1796*	31.4	10.4	*0*.*0025*	0
rs6754561	*SERPINE2*	2	224,839,696	C	0.30	-29.6	12.2	*0*.*0151*	-23.0	19.3	*0*.*2350*	-11.4	26.8	*0*.*6707*	-25.6	9.6	*0*.*0077*	0
rs11926758	*RARB*	3	25,552,252	G	0.94	52.5	22.9	*0*.*0219*	51.3	37.7	*0*.*1734*	48.8	48.1	*0*.*3119*	51.7	18.1	*0*.*0044*	0
rs11716871	*TP63*	3	189,582,501	A	0.92	-55.4	19.1	*0*.*0037*	-32.5	34.0	*0*.*3404*	-36.1	47.2	*0*.*4444*	-48.4	15.7	*0*.*0021*	0
rs4712047	*SIRT5*	6	13,590,185	A	0.66	34.0	11.2	*0*.*0024*	9.9	22.7	*0*.*6622*	27.9	32.3	*0*.*3882*	29.2	9.6	*0*.*0023*	0
rs2722322	*SFRP4*	7	37,948,714	A	0.15	51.7	15.4	*0*.*0008*	36.8	24.3	*0*.*1298*	16.3	35.3	*0*.*6437*	43.7	12.2	*0*.*0003*	0
rs17172023	*GLI3*	7	42,245,499	C	0.78	36.4	13.8	*0*.*0082*	25.2	24.4	*0*.*3034*	10.2	33.8	*0*.*7644*	31.1	11.3	*0*.*0060*	0
rs1049337	*CAV1*	7	116,200,587	C	0.70	33.6	11.6	*0*.*0038*	28.1	19.8	*0*.*1562*	-10.3	29.3	*0*.*7254*	27.8	9.5	*0*.*0034*	0
rs2707469	*WNT16*	7	120,976,886	A	0.83	34.2	14.3	*0*.*0168*	23.6	25.9	*0*.*3611*	34.5	39.1	*0*.*3787*	32.0	11.9	*0*.*0073*	0
rs12549242	*EYA1*	8	72,216,430	C	0.14	-38.6	16.1	*0*.*0167*	-53.8	24.3	*0*.*0267*	-72.2	43.4	*0*.*0972*	-45.8	12.8	*0*.*0004*	0
rs2812427	*DLG5*	10	79,553,236	A	0.67	33.3	11.2	*0*.*0029*	14.8	19.8	*0*.*4548*	63.1	28.5	*0*.*0274*	32.4	9.2	*0*.*0004*	0
rs1994450	*PDGFD*	11	103,797,349	A	0.13	-41.9	18.0	*0*.*0201*	-64.3	29.3	*0*.*0283*	2.1	38.9	*0*.*9573*	-41.3	14.3	*0*.*0038*	0
rs12708369	*NCOR2*	12	124,875,577	C	0.56	25.1	11.5	*0*.*0291*	46.0	20.7	*0*.*0263*	-9.1	30.1	*0*.*7626*	26.1	9.5	*0*.*0062*	13
rs11865499	*KAT8*	16	31,132,250	A	0.69	33.7	11.3	*0*.*0029*	20.4	19.9	*0*.*3061*	6.2	28.9	*0*.*8304*	27.9	9.3	*0*.*0027*	0
rs1880756	*CRHR1*	17	43,826,666	C	0.58	-26.5	10.6	*0*.*0122*	-28.6	19.4	*0*.*1418*	-5.4	27.8	*0*.*8472*	-24.8	8.8	*0*.*0049*	0
rs948589	*SMAD4*	18	48,586,184	A	0.91	-47.2	19.0	*0*.*0131*	-56.4	34.4	*0*.*1013*	-78.5	50.7	*0*.*1227*	-52.2	15.8	*0*.*0010*	0
rs2425024	*MMP24*	20	33,844,938	A	0.66	25.0	11.3	*0*.*0274*	23.9	19.4	*0*.*2184*	55.7	26.9	*0*.*0390*	28.4	9.2	*0*.*0020*	0
rs6061580	*CDH4*	20	60,058,986	C	0.92	-60.4	22.3	*0*.*0067*	-41.5	37.3	*0*.*2657*	-7.6	47.0	*0*.*8717*	-48.7	17.7	*0*.*0060*	0
rs2051179	*RUNX1*	21	36,326,553	A	0.45	-32.0	10.9	*0*.*0032*	-15.7	18.8	*0*.*4038*	-16.1	25.9	*0*.*5336*	-26.6	8.8	*0*.*0026*	0
rs730265	*CLDN14*	21	37,871,886	A	0.15	-25.8	15.6	*0*.*0973*	-41.8	24.2	*0*.*0837*	-55.2	33.7	*0*.*1020*	-33.7	12.2	*0*.*0057*	0
rs2871029	*CLDN5*	22	19,513,930	A	0.14	31.4	15.1	*0*.*0375*	66.5	27.6	*0*.*0161*	-10.3	40.0	*0*.*7969*	34.6	12.6	*0*.*0060*	24
rs5749524	*TIMP3*	22	33,224,285	C	0.89	49.7	17.0	*0*.*0035*	22.4	29.3	*0*.*4452*	58.5	38.1	*0*.*1259*	44.9	13.7	*0*.*0011*	0

**Table 3:** Replication findings for the best SNP of the top 25 genes. Chr: chromosome; EA: effect allele; EAF: effect allele frequency; β (standard error, SE): per-allele effect on FVC (ml); Repl P: one-side replication p-value, calculated and reported only for estimates in the same direction as the original ones; I^2^: between-study heterogeneity; Imp R^2^ = imputation quality R^2^ (for CHARGE and SpiroMeta: average imputation R^2^ across studies)

SNP	Gene	Chr	EA	EAF	STAGE 1meta-analysis(N = 7,749)			STAGE 2								
								CHARGE and SpiroMeta meta-analysis(N = 46,103—Adults)					ALSPAC(N = 5,062—Children)			
					β	SE	*P*	β	SE	*Repl P*	I^2^ (%)	Imp R^2^	β	SE	*Repl P*	Imp R^2^
rs2820472	*WLS*	1	C	0.70	26.5	9.6	*0*.*0055*	0.7	4.7	*0*.*444*	35	0.92	1.6	8.3	*0*.*423*	0.97
rs832169	*PKP1*	1	A	0.17	36.9	12.0	*0*.*0021*	-7.0	4.9	*/*	23	0.85	-2.1	8.3	/	0.94
rs7527525	*ACTN2*	1	C	0.33	28.1	9.5	*0*.*0032*	-4.2	4.6	/	24	0.71	11.2	7.2	*0*.*059*	0.90
rs3905417	*CTNNA2*	2	A	0.23	31.4	10.4	*0*.*0025*	2.5	5.2	*0*.*312*	0	0.95	13.2	9.0	*0*.*071*	0.99
rs6754561	***SERPINE2***	2	C	0.30	-25.6	9.6	*0*.*0077*	-7.1	3.9	*0*.036*	0	0.96	-12.0	7.1	*0*.045*	1.00
rs11926758	*RARB*	3	G	0.94	51.7	18.1	*0*.*0044*	-4.1	7.4	*/*	26	0.98	3.1	12.2	*0*.*401*	0.99
rs11716871	*TP63*	3	A	0.92	-48.4	15.7	*0*.*0021*	17.8	7.5	*/*	0	0.86	-14.4	12.5	*0*.*125*	0.98
rs4712047	*SIRT5*	6	A	0.66	29.2	9.6	*0*.*0023*	0.6	4.7	*0*.*447*	18	0.72	-4.7	8.5	*/*	0.70
rs2722322	*SFRP4*	7	A	0.15	43.7	12.2	*0*.*0003*	-1.7	5.1	*/*	18	0.94	12.0	8.8	*0*.*088*	1.00
rs17172023	*GLI3*	7	C	0.78	31.1	11.3	*0*.*0060*	-9.6	5.3	*/*	27	0.84	8.4	10.0	*0*.*202*	0.75
rs1049337	*CAV1*	7	C	0.70	27.8	9.5	*0*.*0034*	-4.5	5.1	*/*	35	0.69	0.3	7.4	*0*.*484*	1.00
rs2707469	***WNT16***	7	A	0.83	32.0	11.9	*0*.*0073*	10.0	5.2	*0*.026*	6	0.92	11.8	9.4	*0*.*105*	0.90
rs2812427	*DLG5*	10	A	0.67	32.4	9.2	*0*.*0004*	4.5	4.1	*0*.*138*	0	0.95	2.1	7.1	*0*.*382*	1.00
rs1994450	*PDGFD*	11	A	0.13	-41.3	14.3	*0*.*0038*	-1.7	5.5	*0*.*380*	0	0.76	-10.7	9.6	*0*.*132*	0.79
rs12708369	***NCOR2***	12	C	0.56	26.1	9.5	*0*.*0062*	NA^1^	NA^1^	NA^1^	38	0.38	26.9	7.6	*0*.0002**	0.78
rs11865499	*KAT8*	16	A	0.69	27.9	9.3	*0*.*0027*	4.2	4.6	*0*.*181*	30	0.84	10.6	7.5	*0*.*078*	1.00
rs1880756	*CRHR1*	17	C	0.58	-24.8	8.8	*0*.*0049*	-5.0	4.0	*0*.*108*	15	0.96	2.9	7.0	*/*	1.00
rs948589	*SMAD4*	18	A	0.91	-52.2	15.8	*0*.*0010*	8.8	6.7	*/*	0	0.96	-14.7	12.2	*0*.*114*	1.00
rs2425024	*MMP24*	20	A	0.66	28.4	9.2	*0*.*0020*	3.3	4.0	*0*.*205*	0	0.96	-11.3	7.1	*/*	1.00
rs6061580	*CDH4*	20	C	0.92	-48.7	17.7	*0*.*0060*	9.0	8.6	*/*	3	0.73	2.6	13.9	*/*	0.92
rs2051179	*RUNX1*	21	A	0.45	-26.6	8.8	*0*.*0026*	-3.8	3.8	*0*.*159*	26	0.94	-5.9	6.7	*0*.*188*	0.97
rs730265	*CLDN14*	21	A	0.15	-33.7	12.2	*0*.*0057*	-3.0	7.2	*0*.*338*	20	0.50	8.0	8.0	*/*	0.99
rs2871029	*CLDN5*	22	A	0.14	34.6	12.6	*0*.*0060*	-0.7	5.8	*/*	47	0.90	6.0	9.7	*0*.*269*	1.00
rs5749524	*TIMP3*	22	C	0.89	44.9	13.7	*0*.*0011*	2.0	6.0	*0*.*371*	0	0.94	1.4	10.4	*0*.*448*	1.00

* Nominal significance (*p*<0.05)

** Significance after Bonferroni correction (*p*<0.002)

^1^ Results not available: the SNP had a very low average imputation R^2^ (0.38) and no proxies (LD R^2^>0.80) were available

*NCOR2*-rs12708369 replicated in ALSPAC children with an effect of 26.9 ml/allele (95% confidence interval: 12.0 to 41.8) and a p-value well below Bonferroni correction (*p* = 0.0002). The estimate was very similar to that of Stage 1 (26.1; 7.5 to 44.7), suggesting a relatively stronger effect in children given their lower FVC, although the confidence intervals are wide and conclusions as to a difference in effect sizes cannot be deduced. In line with this, the proportion of FVC residual variance explained by this SNP was much higher in children than in adults from Stage 1, 0.65% vs. 0.11%. Replication of *NCOR2*-rs12708369 could not be performed in adults because of low imputation quality (imputation R^2^ = 0.4) and no proxy available. Using publicly available epigenomic profiling data (ChIP-seq) from ENCODE[26] via the UCSC Genome Browser (http://genome.cse.ucsc.edu), we found that the intronic variant *NCOR2*-rs12708369 is in a region with regulatory function in lung tissue. The SNP is located within a DNase I hypersensitivity site, in a strong enhancer element with histone mark H3K27ac indicating active chromatin in lung fibroblasts. Unfortunately neither *NCOR2*-rs12708369 nor any proxy could be tested in the lung eQTL analysis due to failed imputation quality control.

*SERPINE2*-rs6754561, a variant located 133 bp downstream from the gene, replicated at nominal level in adults from the CHARGE and SpiroMeta consortia (-7.1 ml/allele; *p* = 0.036), where there was no heterogeneity across the 24 studies (I^2^ = 0%), and ALSPAC children (-12.0 ml/allele; *p* = 0.045). The proportion of FVC residual variance explained was only 0.01% in adults, but 0.11% in children (0.09% in adults from Stage 1). *SERPINE2*-rs6754561 did not show association with the expression of *SERPINE2* or any nearby genes in the lung eQTL dataset.

The intronic variant *WNT16*-rs2707469 replicated at nominal level in adults (10.0 ml/allele; *p* = 0.026; I^2^ = 6%), but not in children (11.8 ml/allele; *p* = 0.105). The proportion of FVC residual variance explained was only 0.01% in adults from the CHARGE and SpiroMeta consortia (0.10% in Stage 1). This variant is in a conserved region and is located in a DNase I hypersensitivity site in lung fibroblasts. *WNT16*-rs2707469 was not associated with *WNT16* expression but showed suggestive evidence of an effect on a nearby gene, *CPED1*, with the FVC-lowering allele G associated with higher *CPED1* mRNA expression levels (*p* = 0.087; I^2^ = 0%; S2 Fig). We investigated this further and found that the effect on *CPED1* expression was stronger (*p* = 0.004; I^2^ = 0%) for a SNP in high LD with *WNT16*-rs2707469 (R^2^ = 0.94), rs2536166 (S2 Fig).

## Discussion

By testing the association of FVC with genes related to lung development, we have identified a new gene, *NCOR2*, in the retinoic acid signalling pathway pointing to a role of vitamin A metabolism in the regulation of FVC. Our study also provides support for *SERPINE2*, a gene which has previously shown weak evidence of association with FVC, and suggests *WNT16* as a promising candidate requiring further investigation.

*NCOR2* (nuclear receptor corepressor 2), also known as *SMRT* (silencing mediator of retinoid and thyroid hormone), is a potent regulator of retinoid and thyroid hormone signalling. Nuclear receptors are ligand-activated transcription factors that regulate many developmental and physiological processes. Retinoic acid is the biologically active metabolite of vitamin A (retinol) which has a well described role in organogenesis and epithelial homeostasis directing growth, patterning and differentiation of many organs including the lung[27]. *NCOR2* is a transcriptional “platform” protein that acts as a repressive co-regulatory factor for multiple transcription factor pathways. Publicly available data retrieved from BioGPS[21] (Human U133A/GNF1H Gene Atlas database) show that the expression of *NCOR2* in the adult lung is very high and that the gene is also expressed in foetal lung. In this study we found an association of *NCOR2* (rs12708369) with FVC in adults, which strongly replicated in children. Replication in adults from the CHARGE and SpiroMeta consortia could not be performed due to low imputation quality and no data on proxies available either. The *NCOR2-*rs12708369 intronic variant is in a strong transcriptional enhancer element in lung fibroblasts and may therefore affect gene expression levels[28], although we were not able to test this due to the same problem of low imputation quality in the Lung eQTL dataset. The replication of *NCOR2* in children and the known central developmental roles of retinoic acid and thyroid hormone signalling during alveologenesis[29] suggest that this gene may influence lung growth and ultimately FVC. Although retinoic acid has also been postulated to have a role in ongoing alveolar maintenance and regeneration[30], in our study the *NCOR2-*rs12708369 effect in adults could be estimated only in Stage 1 mostly based on 31-year olds, so potential effects on FVC decline would not have been detected. Interestingly, another related gene, the *RARB* encoding the retinoic acid receptor beta, was selected in Stage 1, although it could not be replicated possibly due to the low minor allele frequency of its selected SNP (rs11926758; MAF = 0.06). This gene has been previously associated with measures of airway obstruction in adults and children (FEV_1_/FVC)[31, 32], and in infants (V’maxFRC)[33]. Overall our findings point to a role of vitamin A/thyroid metabolism in the regulation of FVC, and suggest the importance of further research investigating genes in related pathways as well as gene-environment interactions with vitamin A intake.

*SERPINE2* is a member of a gene family encoding serpins, highly conserved proteins that help maintain tissue integrity by controlling the activity of proteases in diverse biological processes, in particular by inhibiting serine proteases such as trypsin. *SERPINE2* has a known link to airway obstruction, with strong evidence of association with COPD[34] and some evidence of association with childhood asthma[35]. Our findings support an association with a marker of lung restriction too, FVC, in both adults and children, in line with previous findings of an association with FVC in children that could not be replicated[36]. *SERPINE2-*rs6754561 showed no effect on the expression of *SERPINE2* or nearby genes in the lung. However, although the Lung eQTL dataset represents the largest eQTL mapping study of human lung samples currently available, weak to moderate effects on gene expression may not have been detected due to insufficient statistical power. Cellular heterogeneity in lung tissue may also impair the detection of cell type-specific eQTL[37].

We also found suggestive evidence of an association of *WNT16* with FVC in adults. *WNT16* belongs to a family of genes encoding 19 Wnt ligands, secreted signalling proteins involved in many developmental processes. Although Wnts are critical for normal lung development[18, 38], Wnt16 has not been previously studied in relation to lung function and disease. In addition to lung development, evidence from mouse models suggests that Wnt16 plays a role in tissue repair[39] and in the response to cellular damage[40]. The *WNT16*-rs2707469 intronic variant is in a conserved region with regulatory function in lung fibroblasts. This variant showed no eQTL effect on *WNT16* in the lung, but an effect on a nearby gene, *CPED1* (cadherin-like and PC-esterase domain containing 1). CPED1 has both a cadherin-like domain, thought to have a carbohydrate binding function, and a PC-esterase domain, predicted to modify cell surface biomolecules like glycoproteins. It is possible that Wnt16, which is a glycoprotein containing carbohydrates, could bind to, and/or be modified by, CPED1.

By focusing on genetic pathways related to lung development, which represent highly plausible candidates for low FVC, our study identifies a novel gene and proposes two further promising candidates which had not been identified in the previous GWA meta-analysis[9]. This shows how a comprehensive hypothesis-driven approach can complement hypothesis-free GWA analyses in identifying variants which failed to reach the strict significance level needed to protect against false positives in genome-wide investigations (typically 5x10^-8^). However, we did miss the association of one of the genes we tested, *PTCH1*, a gene which has shown association with FVC in the previous GWA meta-analysis[9] and had been identified before as associated with FEV_1_/FVC[32, 41]. The three SNPs previously identified in *PTCH1* had non-significant p-values in our Stage 1 analysis, most likely due to their relatively low minor allele frequency (MAF between 0.08 and 0.10), which made our analysis underpowered to detect them.

In conclusion, this study identifies *NCOR2* as a new gene for FVC, indicating the importance of further research into the role of vitamin A intake/supplementation and its interactions with related genes in the regulation of FVC. Our findings also suggest other biological pathways as promising candidates for future investigation. We might expect genes involved in lung development to show stronger effects in childhood, and the relatively large replication estimate of the effect of *NCOR2-*rs12708369 in children seems to support this. We speculate that future investigation of genes involved in lung development in larger samples of children and young adults could identify further genetic variants associated with FVC through their effect on lung growth and maximum level attained.

## Supporting Information

S1 Fig Regional association plots for *NCOR2* rs12708369, *SERPINE2* rs6754561 and *WNT16* rs2707469. (DOC)

S2 Fig Forest plots for the meta-analyses of lung gene expression levels of *CPED1* associated with *WNT16* variants. (DOC)

S1 Table Stage 1 study-specific and meta-analysis results for all the 24,728 SNPs in the 403 genes. (XLSX)

S2 Table Spirometry methods for studies in Stage 1. (DOC)

S3 Table Genotyping and imputation methods for studies in Stage 1. (DOC)
