# Supplementary material for: Association of Forced Vital Capacity with the Developmental Gene NCOR2
Source: PLoS One. 2016 Feb 2;11(2):e0147388. doi: 10.1371/journal.pone.0147388 (PMC4737618; doi:10.1371/journal.pone.0147388)
Supplement: S1 Fig — (DOC) [file pone.0147388.s001.doc]

**S1 Figure. Regional association plots for *NCOR2* rs12708369, *SERPINE2* rs6754561 and *WNT16* rs2707469**. The plots show (-log_10_) p-values for association with FVC from Stage 1, for all SNPs in the region ordered by chromosome position. The colour reflects the correlation (linkage disequilibrium r^2^) of each SNP with the lead SNP. The plots have been produced using SNAP (<http://www.broadinstitute.org/mpg/snap>)

1. ***NCOR2* rs12708369**

**
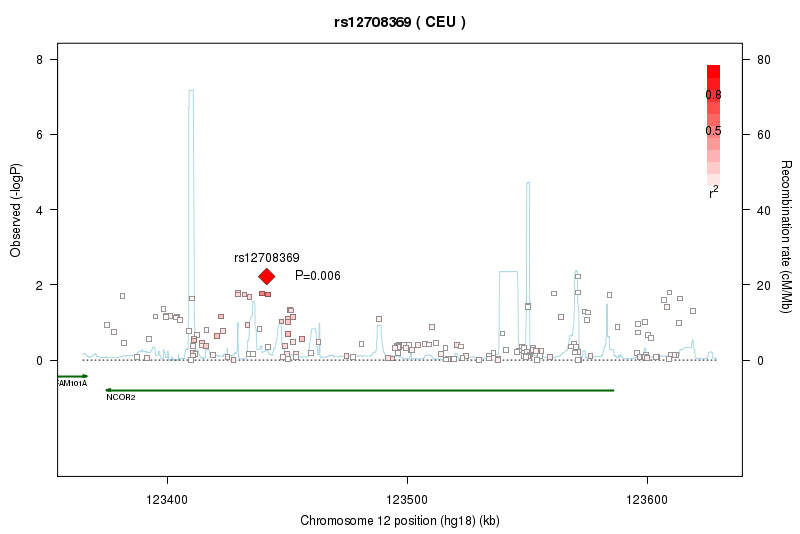
**

1. ***SERPINE2* rs6754561**

**
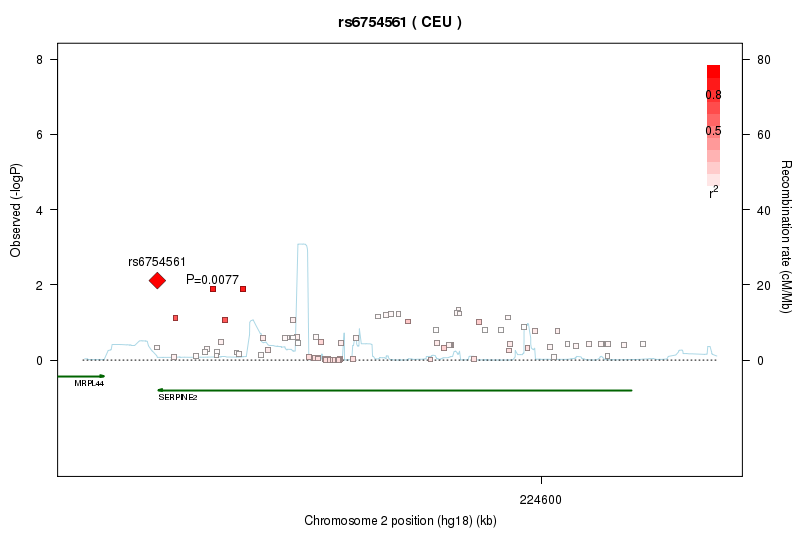
**

1. ***WNT16* rs2707469**

**
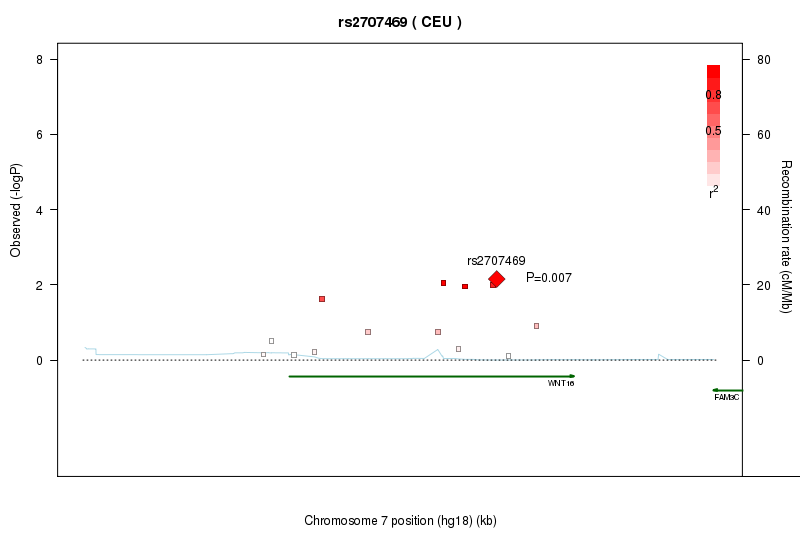
**
