# Supplementary material for: Association of Forced Vital Capacity with the Developmental Gene NCOR2
Source: PLoS One. 2016 Feb 2;11(2):e0147388. doi: 10.1371/journal.pone.0147388 (PMC4737618; doi:10.1371/journal.pone.0147388)
Supplement: S2 Fig — (DOC) [file pone.0147388.s002.doc]

**S2 Figure. Forest plots for the meta-analyses of lung gene expression levels of *CPED1* associated with *WNT16* variants.** a) *WNT16*-rs2707469, allele G; b) *WNT16*-rs2536166, allele A (R^2^=0.94 with rs2707469). Expression is for probeset 100310336_TGI_at, and gene expression levels are adjusted for age, gender and smoking status. The size of the squares is proportional to the precision of the estimates for each center, with the horizontal lines indicating their 95% conﬁdence intervals. The pooled estimate is represented by the centre of the diamond, with the lateral tips indicating its 95% conﬁdence interval. The solid vertical line is the line of no effect

1. ***WNT16* rs2707469**

*p* = 0.087

1. ***WNT16* rs2536166**

*p* = 0.004
