# Supplementary material for: Association of Forced Vital Capacity with the Developmental Gene NCOR2
Source: PLoS One. 2016 Feb 2;11(2):e0147388. doi: 10.1371/journal.pone.0147388 (PMC4737618; doi:10.1371/journal.pone.0147388)
Supplement: S2 Table — (DOC) [file pone.0147388.s004.doc]

**S2 Table. Spirometry methods for studies in Stage 1**. ATS: American Thoracic Society

| **Study** | **Spirometer** | **Methods** |
| --- | --- | --- |
| NFBC1966 | Vitalograph P-model spirometer (Vitalograph Ltd., Buckingham, UK) | - Spirometric manoeuvres performed three times, but repeated if coefficient of variation between two maximal readings >4%  - Spirometer calibrated regularly, with a volumetric accuracy of ±2% or ±50 mL, whichever was greater |
| ECRHS | Biomedin water-sealed spirometer (Biomedin, Padova, Italy): 9 centres  SensorMedics spirometer (Sensormedics, Yorba Linda, USA): 4 centres  Jaeger Pneumo Lab spirometer (Jaeger, Würzburg, Germany): 3 centres | - Spirometric manoeuvres performed in the sitting position with nose clips; up to nine attempts performed to provide at least two technically acceptable manoeuvres  - Volume signal of equipment verified on a daily basis using calibrated syringes (2 or 3 L)  - Spirometry data included only if forced expiratory manoeuvre satisfactory and compliant with ATS criteria |
| EGEA | First survey, EGEA 1: Biomedin water-sealed spirometer (Biomedin, Padova, Italy) and Jaeger Pneumotach (Jaeger, Würzburg, Germany)  Second survey, EGEA 2: Spirodyn flow-volume spirometer (SPIRODYN’R; SAS Dyn'R, Aix-en-Provence, France) | - Spirometry manoeuvres performed at least three times in the sitting position with nose clips; a minimum of two acceptable forced expiratory flows complying with ATS criteria were obtained  - Spirometry data included only if forced expiratory manoeuvre satisfactory and compliant with ATS criteria |
