# Supplementary material for: Association of Forced Vital Capacity with the Developmental Gene NCOR2
Source: PLoS One. 2016 Feb 2;11(2):e0147388. doi: 10.1371/journal.pone.0147388 (PMC4737618; doi:10.1371/journal.pone.0147388)
Supplement: S3 Table — (DOC) [file pone.0147388.s005.doc]

**S3 Table. Genotyping and imputation methods for studies in Stage 1**

| **Study** | **Genotyping platform** | **QC filters and reasons for exclusion of participants** | **N. of genotyped autosomal SNPs passing QC** | **Imputation software** | **NCBI Build for imputation reference (HapMap CEU)** |
| --- | --- | --- | --- | --- | --- |
| NFBC1966 | Illumina CNV 370 Duo | **SNPs**:  Call rate <95%  HWE p-value <10^-4^  MAF <1%  **Participants**:  Contaminated sample  Sex mismatch  Cryptic relatedness  Withdrew consent | 328,007 | IMPUTE v1.0^1^ | Build 37 (1000 Genomes) |
| ECRHS | Illumina 610k | **SNPs**:  Call rate <95%  HWE p-value <10^-4^  MAF <1%  **Participants**:  Sex mismatch  Cryptic relatedness  High X chromosomal heterozygosity for males | 582,892 | MACH 1.0^2^ | Build 36, release 22 |
| EGEA | Illumina 610k | **SNPs**:  Call rate <97%,  HWE p-value <10^-4^  MAF <5%  **Participants:**  Contaminated sample  Call rate <97%  Sex mismatch  Cryptic relatedness  Non-European descent | 513,460 | MACH 1.0^2^ | Build 36, release 22 |

**^1^** Howie BN, Donnelly P, Marchini J (2009) A flexible and accurate genotype imputation method for the next generation of genome-wide association studies. PLoS Genet 5: e1000529; **^2^** Li Y, Abecasis GR (2006) Mach 1.0: Rapid Haplotype Reconstruction and Missing Genotype Inference. Am J Hum Genet S79: 2290
